# Spike culture derived wheat (*Triticum aestivum* L.) variants exhibit improved resistance to multiple chemotypes of *Fusarium graminearum*

**DOI:** 10.1371/journal.pone.0226695

**Published:** 2019-12-19

**Authors:** Chen Huang, Manu P. Gangola, Seedhabadee Ganeshan, Pierre Hucl, H. Randy Kutcher, Ravindra N. Chibbar

**Affiliations:** 1 Department of Plant Sciences, College of Agriculture and Bioresources, Campus Drive, University of Saskatchewan, Saskatoon, Saskatchewan, Canada; 2 Crop Development Centre, College of Agriculture and Bioresources, Campus Drive, University of Saskatchewan, Saskatoon, Saskatchewan, Canada; Institute of Genetics and Developmental Biology Chinese Academy of Sciences, CHINA

## Abstract

Fusarium head blight (FHB) in wheat (*Triticum aestivum* L.), predominantly caused by *Fusarium graminearum*, has been categorized into three chemotypes depending on the major mycotoxin produced. The three mycotoxins, namely, 3-acetyldeoxynivalenol (3-ADON), 15-acetyldeoxynivalenol (15-ADON) and nivalenol (NIV) also determine their aggressiveness and response to fungicides. Furthermore, prevalence of these chemotypes changes over time and dynamic changes in chemotypes population in the field have been observed. The objective of this study was to identify spike culture derived variants (SCDV) exhibiting resistance to multiple chemotypes of *F*. *graminearum*. First, the optimal volume of inoculum for point inoculation of the spikelets was determined using the susceptible AC Nanda wheat genotype. Fifteen μL of 10^5^ macroconidia/mL was deemed optimal based on FHB disease severity assessment with four chemotypes. Following optimal inoculum volume determination, five chemotypes (Carman-NIV, Carman-705-2-3-ADON, M9-07-1-3-ADON, M1-07-2-15-ADON and China-Fg809-15-ADON) were used to point inoculate AC Nanda spikelets to confirm the mycotoxin produced and FHB severity during infection. Upon confirmation of the mycotoxins produced by the chemotypes, 55 SCDV were utilized to evaluate FHB severity and mycotoxin concentrations. Of the 55 SCDV, five (213.4, 244.1, 245.6, 250.2 and 252.3) resistant lines were identified with resistance to multiple chemotypes and are currently being utilized in a breeding program to develop wheat varieties with improved FHB resistance.

## Introduction

Fusarium head blight (FHB) is one of the major fungal diseases of wheat (*Triticum aestivum* L.), caused mainly by *Fusarium* species among which *Fusarium graminearum* Schwabe [teleomorph *Gibberella zeae* (Schwein.) Petch] [1] is the most predominant in North America [2]. High humidity and warm temperatures [3, 4] in the presence of abundant natural inoculum favor FHB development in wheat during grain-filling stages [5–7] restricting grain development, producing light shriveled grains and reducing grain yield [8, 9]. FHB epidemics during 1993 (Manitoba in Canada; Minnesota, North Dakota and South Dakota in the USA), 1996 (Ontario, Canada), and 1991–1997 (USA) caused >US$ 1 billion, >CA$ 100 million, and >US$ 1.3 billion losses, respectively, to wheat production and export [10].

FHB also diminishes crop value as the infected grains accumulate the trichothecene group of mycotoxins predominantly, Deoxynivalenol (DON), and Nivalenol (NIV) (S1 Fig) [11]. DON producing chemotypes also accumulate its two acetylated forms, 3-Acetyldeoxynivalenol (3-ADON), and 15-Acetyldeoxynivalenol (15-ADON) establishing two subgroups of DON [12]). Recently, *F*. *graminearum* strains producing a novel type A trichothecene mycotoxin NX-2 (3α-acetoxy,7α,15-dihydroxy-12,13-epoxytrichothec-9-ene) has been reported from the Midwestern USA [13] and in Ontario, Canada [14], but it is not widely distributed in Canada [15]. NX-2 has a structure similar to 3-ADON, but lacks keto group at C-8 position, and the deacetylated form is NX-2 [16].

Although comprehensive FHB resistance mechanism in plants is not yet understood, detoxification of mycotoxin is one of the widely studied mechanism in wheat and other cereal crops. DON or NIV in plants can be glucosylated in an UDP-Glucosyltransferase (EC 2.4.1.x) catalyzed reaction into Deoxynivalenol-3-glucoside (D3G) or Nivalenol-glucoside, respectively, which is non-toxic to plants [17, 18]. The mycotoxin (DON, 3-ADON, 15-ADON and NIV) also act as virulence factors thus facilitating post-infection or post-inoculation disease spread within the wheat spike [9]. The 3-ADON chemotypes, accumulate two-fold higher mycotoxin, thus producing higher FHB severity compared to 15-ADON and NIV chemotypes [19]), and have become more prevalent in North America, Asia and Europe [20, 21].

The mycotoxins are highly stable compounds that do not degrade at elevated temperatures, or during the milling, storage or food processing stages [22]. The consumption of food or feed contaminated with DON, acetylated products and NIV adversely affect gastrointestinal and immune-modulatory systems in humans and animals [23]. Consequently, in 37 countries, the highest permitted DON concentration vary from 200–2000 μg kg^-1^ in cereals [24], including Asian countries (500–2000 μg kg^-1^) [25], European countries (200–1750 μg kg^-1^) [26], and Canada (2000 μg kg^-1^) [27]. Similarly, attempts at restricting limits of NIV have also been recognized since it is more toxic to animal and human health [28, 29] and regulations across the globe are aiming to limit concentrations of NIV from 20–60 μg kg^-1^ in France to 584–1780 μg kg^-1^ in China [30].

Considering the existence of the *Fusarium graminearum* species complex with the different chemotypes and numerous mycotoxins produced, breeding for FHB resistance in wheat has been challenging. It is now well-established that FHB resistance is a complex quantitative trait influenced by genotype, fungal chemotype, environment and agricultural/management practices [11]. Similarly, selection pressure and environment may affect chemotype diversity of *F*. *graminearum* population and compounding expression of field resistance in wheat from year to year. This is due to the occurrence of genetic diversity in important genes of *F*. *graminearum* and sexual recombination which in turn affect population architecture of a region and the proportion of distinct chemotypes [31]. The mycotoxin, on the other hand, determines the virulence of a chemotype [32] influencing its occurrence and competition with other chemotypes to infect a crop. Consequently, a shift in 15-ADON chemotype population has been documented in western Canada, wherein more than a fourteen-fold increase in frequency of 3-ADON chemotypes was observed from 1998 to 2004 [20]. Studies on the distribution of *F*. *graminearum* species complex in various countries have indicated a significant impact of environmental or climatic factors on *F*. *graminearum* population dynamics [reviewed in 33]. Furthermore, agricultural practices such as zero or minimum tillage have also contributed to shifts in genetic diversity in *F*. *graminearum* population by providing more stubble for fungal survival [31].

The quantitative nature of FHB resistance in wheat combined with the occurrence of chemotypes and shifts in chemotype populations of *F*. *graminearum*, implies a multi-faceted approach to develop FHB resistance. While breeding efforts are on-going and allow for continual release of new resistant varieties to keep pace with the dynamic changes in the *F*. *graminearum* chemotype population, complementary technological approaches would also greatly contribute to screening for and developing FHB resistance. Thus, developing wheat genotypes for resistance to multiple chemotypes of *F*. *graminearum* would be valuable. Therefore, the objective of the present study is to identify spike culture derived wheat variants (SCDV) [34, 35] resistant to multiple *F*. *graminearum* chemotypes which can be utilized in breeding programs to develop wheat genotypes with broad spectrum FHB resistance. The SCDV was previously developed by ethyl methane sulfonate (EMS) mutagenesis [34] and shown to exhibit a range of FHB disease severity using an immature spike culture screening method [35].

## Materials and methods

### Plant material and *Fusarium graminearum* strains

Wheat (*Triticum aestivum* L.) cv AC Nanda [36] and landrace Sumai-3 were used as FHB susceptible and resistant controls, respectively. Fifty-five spike culture derived variants (SCDV) obtained from an EMS mutagenized population from wheat were used to screen for FHB resistance to five different *Fusarium graminearum* Schwabe [telomorph: *Gibberella zeae* (Schw.) Petch] strains (M9-07-1, M1-07-2, Carman-NIV, China-Fg809, and Carman-705-2) procured from the former Cereal Research Centre (Agriculture and Agri-Food Canada, Winnipeg, MB, Canada). The Fusarium strains were regrown in liquid culture to the required macroconidia concentrations as previously described [35].

To collect spikes for immature spike cultures, wheat plants were grown in a greenhouse (University of Saskatchewan, Saskatoon, SK, Canada) till the onset of the heading. The immature spikes were excised from the plants and maintained in *in vitro* spike culture media (pH 6.2) containing sucrose (50 g/L), L-glutamine (0.4 g/L) and morpholinoethanesulfonic acid (0.5 g/L) for five days as previously described [37, 38]. Spikes were used for inoculation and disease severity assessment calculated as FHB severity (%) per spike = (number of symptomatic or infected spikelets × 100) / total number of spikelets as previously described [39].

### Determination of optimal inoculum volume for inoculating to spikelets

Spikes from AC Nanda and Sumai-3 were point inoculated with varying volume (10, 15 and 20 μL) of inoculum at 10^5^ macroconidia/mL concentration with different chemotypes, covered with moist plastic bags for three days to facilitate fungal infection and placed in a Sanyo versatile test chamber (Sanyo-MIR351H, Canada) at 16/8 h day/night cycle with 26/24°C temperature and disease severity determined as previously described [35].

### Point inoculation of spikelets with different chemotypes of *F*. *graminearum*

After the initial experiments indicated that 15 μL volume of inoculum was optimal, spikes from AC Nanda in three replications were independently inoculated with the five strains of *Fusarium graminearum*. The spikes in three replications were collected at 2, 5, 7, 9, and 11 days after inoculation (DAI) in liquid nitrogen, stored at -80°C for mycotoxin extraction. FHB disease severity data was also recorded at 2, 5, 7, 9, and 11 DAI.

### Mycotoxin extraction and detection

The infected wheat spikes from each Fusarium strain were used to extract mycotoxin in an extraction buffer (acetonitrile:water, 84:16, v/v) using a recently optimized method [39]. The extracted mycotoxin was analyzed using a 4000 QTRAP LC-MS/MS system (AB SCIEX, Framingham, MA, USA) equipped with a high-performance liquid chromatography, an electrospray interface and a hybrid triple quadrupole ion trap mass spectrometer. The chromatograms were evaluated using Analyst 1.6.2 software (AB SCIEX, Framingham, MA, USA) [39].

### Identification of SCDV lines resistant against multiple chemotypes

Fifty-five spike culture derived variants (SCDV) were selected based on their FHB reaction from a population of 134 SCDV lines developed in previous study [35]. The selected SCDV lines at M_3_ stage were grown in a greenhouse during May-July 2016 with 16/8 h of day/night cycle with average daily temperature of 27.3/20.6°C and relative humidity of 50.1/70.5%. The immature spikes in three replicates were collected and evaluated for FHB severity at 5, 7, 9, and 11 DAI, and calculated as described [39].

### Statistical analysis

Minitab 16 statistical software (Minitab Inc., Pennsylvania, USA) was used for correlation analysis by calculating Pearson’s correlation coefficient for association of FHB severity to mycotoxin accumulation during FHB progression. ANOVA was performed to evaluate the effect of genotypes and chemotypes on mycotoxin accumulation during FHB progression. To find differences among chemotypes *post-hoc* analysis Tukey’s pairwise comparisons were performed. The box-plot analysis to depict the variation in FHB severity among spike culture derived wheat variants infected with distinct *F*. *graminearum* chemotypes was generated using SigmaPlot version 10 (Systat Software Inc., San Jose, CA, USA).

## Results

### Optimal inoculum volume for point inoculation of spikelets

For point inoculation of spikelets, it is important to deliver an appropriate volume of inoculum. To determine the required optimal volume, an experiment was conducted by injecting 10, 15 and 20 μL of inoculum of strains M9-07-01, M1-07-2, M7-07-1 and Carman-NIV-702 at standardized concentrations of 10^5^ macroconidia/mL into the spikelets of susceptible AC Nanda and tolerant Sumai-3. FHB disease severity was assessed after nine days (**Table 1**). Inoculum volumes of 20 μL were not conducive for point inoculation, as in most instances there was overflow from within the spikelets. When the inoculum was retained within the spikelets, disease progressed rapidly (within seven days) as shown by bleaching, and precluded disease severity assessment. With 10 and 15 μL inoculum volumes, disease severity assessments could be adequately conducted on point-inoculated spikelets nine days after inoculation. The disease severity with highly virulent chemotype, M9-07-1-3ADON, was 65.6% with 10 μL inoculum and 90.72% with 15 μL inoculum on AC Nanda. On Sumai-3, disease severity was 46.1% and 59.8% with 10 and 15 μL inoculum, respectively. With the weakly virulent strain, M1-07-2-15ADON, the disease severity was 44.2% and 65.28% with 10 and 15 μL inoculum, respectively, on AC Nanda. On Sumai-3, the disease severity was 30.8% and 40.7% with 10 and 15 μL inoculum, respectively.

**Table 1:** Influence of inoculum volume on FHB disease severity in point-inoculated spikelets of AC Nanda and Sumai-3.

	Genotypes			
	AC Nanda		Sumai-3	
	Inoculum volume (μL)			
Chemotypes	10	15	10	15
M9-07-1-3ADON	65.6 ± 2.5	90.72 ± 13.13	46.1 ± 4.3	59.8 ± 2.3
M7-07-1-3ADON	52.7 ± 5.4	82.6 ± 2.0	30.3 ± 8.4	37.6 ± 1.3
M1-07-2-15ADON	44.2 ± 1.8	65.28 ± 1.03	30.8 ± 2.4	40.7 ± 2.2
Carman-NIV-702	45.4 ± 9.9	60.3 ± 5.3	36.8 ± 5.9	42.6 ± 3.4

### Confirmation of chemotypes in point-inoculated AC Nanda spikelets

After establishing the required optimal inoculum volume for point-inoculation of spikelets to be 15 μL, the spikes of the susceptible AC Nanda genotype were used to test five strains of *F*. *graminearum* of known chemotype disposition for their mycotoxin production and disease severity in spikelets. Mycotoxin accumulation and FHB severity were determined on 2, 5, 7, 9 and 11 DAI (S2 Fig). Results at 7 and 9 DAI appeared more informative for the mycotoxin accumulation and FHB severity and are presented in **Table 2**.

**Table 2:** Mycotoxins concentration and FHB disease severity in AC Nanda spikelets point-inoculated with different chemotypes at (a) 7 DAI and (b) 9 DAI.

**(a)**						
**7 DAI**	**Mycotoxin concentration (mg/kg)**					**FHB Severity (%)**
	**DON**	**D3G**	**3-ADON**	**15-ADON**	**NIV**	
M1-07-02-15ADON	15.33 ± 0.21	6.93 ± 0.04	0.28 ± 0.01	1.75 ± 0.00	0.03 ± 0.01	50.59 ± 1.52
China Fg809-15ADON	8.91 ± 0.55	5.41 ± 0.16	0.32 ± 0.01	1.59 ± 0.08	0.02 ± 0.01	49.02 ± 5.54
M9-07-01-3ADON	15.42 ± 0.76	8.67 ± 0.04	2.55 ± 0.00	0.00 ± 0.00	0.13 ± 0.00	65.29 ± 10.26
Carman 705-2-3ADON	12.39 ± 0.38	7.44 ± 0.59	2.18 ± 0.09	0.00 ± 0.00	0.11 ± 0.03	65.25 ± 2.75
Carman NIV	0.01 ± 0.00	0.02 ± 0.00	0.06 ± 0.00	0.00 ± 0.00	3.54 ± 0.08	47.565 ± 8.43
**(b)**						
**9 DAI**	**Mycotoxin concentration (mg/kg)**					**FHB Severity (%)**
	**DON**	**D3G**	**3-ADON**	**15-ADON**	**NIV**	
M1-07-02-15ADON	21.96 ± 0.51	11.34 ± 0.14	0.47 ± 0.01	1.73 ± 0.08	0.07 ± 0.01	55.28 ± 0.73
China Fg809-15ADON	11.26 ± 0.23	7.94 ± 0.17	0.28 ± 0.03	0.66 ± 0.04	0.05 ± 0.03	59.98 ± 0.89
M9-07-01-3ADON	16.44 ± 1.02	14.34 ± 0.76	2.42 ± 0.01	0.00 ± 0.00	0.15 ± 0.04	90.72 ± 13.13
Carman 705-2-3ADON	18.24 ± 0.17	12.78 ± 0.25	2.95 ± 0.14	0.00 ± 0.00	0.18 ± 0.01	72.41 ± 5.74
Carman NIV	0.01 ± 0.02	0.05 ± 0.01	0.02 ± 0.02	0.00 ± 0.00	11.29 ± 0.39	60.26 ± 3.74

Carman-NIV, the nivalenol producing chemotype, accumulated mainly nivalenol as expected in the infected spikes with the concentration increasing to 3.54 mg/kg at 7 DAI and 11.29 mg/kg at 9 DAI. DON, D3G and 3-ADON were also detected in trace amounts. FHB severity was 47.6% at 7 DAI and 60.2% at 9 DAI. The two 15-ADON chemotypes, M1-07-2 and China Fg809, accumulated between 0.66 and 1.75 mg/kg of 15-ADON at 7 and 9 DAI and between 8.9 and 22 mg/kg DON at 7 and 9 DAI. Trace amounts of NIV were also detected in the 15-ADON chemotypes. FHB disease severity with the 15-ADON chemotypes ranged from 49 to 60%. The two 3-ADON chemotypes, M9-07-1 and Carman 705–2, showed between 2.4 and 2.95 mg/kg of 3-ADON accumulation at 7 and 9 DAI. DON concentrations ranged from 12.5 to 22 mg/kg, with FHB severity of 65 to 91% at 7 and 9 DAI. Concentrations of NIV ranged from 0.11 to 0.18 mg/kg in 3-ADON chemotype infected spikelets. Both the 3-ADON and 15-ADON chemotypes showed accumulation of D3G in the range of 5.4 to 14.3 mg/kg at 7 and 9 DAI, being significantly (P≤0.001) higher in 3-ADON than 15-ADON at 9 DAI.

Correlation analyses of disease severity and mycotoxin accumulation data at various intervals after inoculation of AC Nanda spikelets indicated significant positive correlation of FHB severity to concentrations of DON (r = 0.659, *P* ≤ 0.001) and 3-ADON (r = 0.439, *P* ≤ 0.01). D3G, the glucoside derivative of DON, also significantly correlated (r = 0.597, *P* ≤ 0.001) with disease severity. Significant positive correlation was observed for DON concentration and D3G (r = 0.931, *P* ≤ 0.001) and 3-ADON (r = 0.5, *P* ≤ 0.05) concentrations. NIV and 15-ADON did not correlate significantly to FHB severity.

### Spike culture derived variants exhibited a range of FHB disease severity

**Fig 1:**
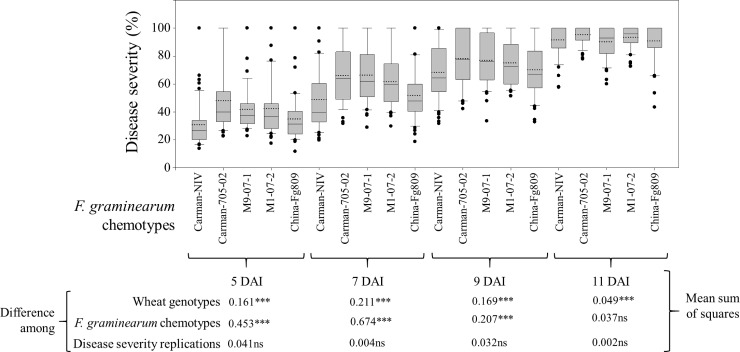
Boxplot analysis summarizing Fusarium head blight (FHB) severity at 5, 7, 9 and 11 days after inoculation (DAI) in the spikes of 55 spike culture derived wheat variants infected with *F*. *graminearum* chemotypes (M9-07-1, M1-07-2, Carman-NIV, China-Fg809, and Carman-705-2) along with Mean Sum of Square values obtained from Analysis of Variance using General Linear Model showing differences for FHB severity among genotypes, chemotypes and replications. The upper and lower error bars represent the non-outlier range of the data set. The box represents the interquartile range (IQR), whereas the middle solid line shows the median value of the data set. The black circles re-present the outliers, calculated as the data points out of the 1.5 times the IQR. Dotted lines in the boxes represent average of the disease severity for the corresponding dataset whereas, *** and ns stand for significant difference at *P* ≤ 0.001 and non-significant, respectively.

The 55 SCDV lines differed significantly in FHB severity (**Fig 1**), however it was less discernible at 5 DAI for all five chemotypes, with several outliers. Tangible disease severity differences started to emerge at 7 DAI. More reliable differences for the five chemotypes were observed at 9 DAI with only few outliers prior to the Q1 quartiles. At 11 DAI, disease severity was skewed towards the upper quartiles into the 100% range precluding within chemotypes inferences. ANOVA analyses also indicated significant (*P* ≤ 0.001) SCDV and chemotypes effects for all DAI on FHB severity (**Fig 1**).

Of the 55 SCDV lines, five lines showed resistance against multiple chemotypes of *F*. *graminearum*. Reduced FHB severity was observed at 5, 7, 9 and 11 DAI for these five lines. As mentioned above, disease severity was more discernible at 7 and 9 DAI, and therefore FHB severity of these five SCDV lines were compared to those of AC Nanda and Sumai-3 spikelets at 7 and 9 DAI (**Table 3**). As expected, AC Nanda exhibited susceptibility to all five chemotypes (**Table 3**). With the tolerant genotype Sumai-3, only the highly virulent chemotype, M9-07-1-3-ADON, caused a disease severity of 59.8%, with the other chemotypes showing reduced disease severity. Generally, the disease severity of the five SCDV lines was comparable to that of Sumai-3 for all five chemotypes.

**Table 3:** Comparison of Fusarium head blight (FHB) at 7 and 9 days after inoculation among the selected spike culture derived wheat variants, AC Nanda (FHB susceptible control) and Sumai-3 (FHB resistant control).

Chemotypes	Days after inoculation	FHB Severity (%)						
		Spike Culture Derived Wheat Variants					AC Nanda	Sumai-3
		213.4	244.1	245.6	250.2	252.3		
M1-07-2-15ADON	7	30.7 ± 2.1	48.4 ± 8.2	39.6 ± 0.6	29.7 ± 2.7	46.3 ± 1.5	50.4 ± 2.1	35.9 ± 1.7
	9	44.9 ± 1.4	51.2 ± 4.2	51.4 ± 5.1	54.4 ± 1.7	53.3 ± 0.9	65.3 ± 1.0	40.7 ± 2.2
China-Fg809-15ADON	7	33.9 ± 7.5	32.5 ± 5.5	32.4 ± 8.9	34.8 ± 12.3	30.5 ± 1.9	49.0 ± 7.8	34.5 ± 3.4
	9	47.3 ± 0.8	43.7 ± 1.2	64.3 ± 5.1	55.9 ± 5.2	43.9 ± 0.9	59.9 ± 1.3	44.6 ± 0.7
M9-07-1-3ADON	7	45.5 ± 11.9	40.0 ± 11.3	45.5 ± 9.4	42.5 ± 11.1	31.8 ± 4.6	65.3 ± 10.3	59.8 ± 2.3
	9	63.6 ± 9.3	44.0 ± 5.7	55.4 ± 7.7	55.6 ± 4.9	56.1 ± 1.5	90.7 ± 13.1	59.8 ± 2.3
Carman-705-2-3ADON	7	44.0 ± 0.0	45.0 ± 8.7	31.7 ± 4.4	48.6 ± 2.0	36.6 ± 2.2	65.3 ± 3.9	26.6 ± 3.6
	9	46.0 ± 2.8	49.9 ± 4.8	47.7 ± 0.2	55.1 ± 0.2	47.3 ± 3.2	73.9 ± 6.0	44.6 ± 0.8
Carman-NIV	7	20.6 ± 7.8	37.3 ± 4.8	50.3 ± 12.3	50.9 ± 7.1	39.5 ± 1.7	42.6 ± 4.9	35.1 ± 2.5
	9	38.6 ± 5.1	52.3 ± 3.9	64.3 ± 3.4	86.0 ± 0.5	48.3 ± 4.7	60.3 ± 5.3	42.6 ± 3.4

## Discussion

A population of 134 ethyl methane sulfonate treated spike culture derived variants (SCDV) lines previously developed from AC Nanda showed significant variation for FHB disease severity ranging from 15 to 100% at 7 DAI when infected with *F*. *graminearum* isolate M7-07-1, a 3-ADON producing chemotype [35]. The present study utilized 55 of those SCDV lines showing the most consistent and contrasting FHB resistance/severity to identify SCDV lines resistant to multiple distinct *F*. *graminearum* chemotypes. Prior to conducting the experiment, it was important to determine the optimal inoculum volume for point-inoculation of the spikelets. Using the susceptible AC Nanda and tolerant Sumai-3 genotypes and four *F*. *graminearum* strains, it was determined that 15 μL was an optimal volume for disease severity assessment (**Table 1**). In a previous study, 15 μL of inoculum at concentrations of 10^5^, 10^4^ and 10^3^ macroconidia/mL were shown to be viable when plated on water-agar with ˃ 99% macroconidia germination [35]. The present study has shown that 15 μL inoculum volume is equally optimal for disease severity assessment.

The determined optimal 15 μL volume inoculum was then used as a standard for point-inoculation of AC Nanda spikelets with five chemotypes of *F*. *graminearum* to confirm mycotoxin production in infected spikelets. The chemotypes used have previously been categorized as 3-ADON producing (M9-07-1 and Carman-705-2), 15-ADON producing (M1-07-2 and China-Fg809) and NIV producing (Carman-NIV) [35, 40]. In the present study, LC-MS/MS was used to detect and quantify the trichothecene mycotoxin produced in the infected spikelets by the five chemotypes. The occurrence of DON and its acetylated forms (3-ADON and 15-ADON) in FHB infected spikelets with the respective chemotypes was not surprising and corroborates findings of previous studies [e.g., 33, 41, 42]. However, the Carman-NIV chemotype produced trace amounts of DON in addition to the major nivalenol mycotoxin, as observed in a previous study [43]. Similar to our studies, 3-ADON chemotypes producing trace amounts of 15-ADON or 15-ADON chemotypes producing trace amounts of 3-ADON have been observed [44–46]. Nonetheless, the detection and quantification of the major mycotoxin associated with the specific chemotype strain was unequivocal.

The virulence of trichothecene mycotoxin in wheat have been demonstrated by trichothecene non-producing mutants, wherein reduced FHB spread was observed [47, 48]. Correlation analyses for DON and FHB severity in the present study also confirm the virulence of the strains used on the susceptible AC Nanda genotype. DON and 3-ADON act as virulent factor to support fungal growth and spread within the infected spike, and reflected in the significant positive correlation to FHB severity. Conversely, NIV and 15-ADON did not correlate to FHB severity significantly due to either absence or presence in trace concentrations in spikes infected with distinct chemotypes. The non-plant toxic D3G form of DON, is glucosylated within plants by UDP-Glucosyltransferase (UGT) encoded by *UGT* genes. Gradual increase in expression during FHB progression within wheat spikes in FHB susceptible genotypes like AC Nanda have been shown [35]. Thus, with increase in DON concentration during FHB progression higher concentration of D3G was observed resulting in significant positive correlation to FHB severity. The lack of correlation between 15-ADON and FHB severity and NIV and FHB severity is due to the low concentrations of these mycotoxin being produced and likely masked by the higher concentration of DON produced.

Spike culture derived variants infected with distinct chemotypes varied significantly for FHB severity. Higher aggressiveness of 3-ADON producing chemotypes compared to 15-ADON chemotypes in this study are in agreement with observations of Puri and Zhong [19]. However, von der Ohe et al. [49] and Liu et al. [50] did not find any significant difference among distinct chemotypes of *F*. *graminearum* for aggressiveness but observed higher accumulation of DON in 3-ADON infected grains. Interestingly, Serajazari et al. [51] observed higher aggressiveness of 3-ADON chemotypes compared to 15-ADON chemotypes for Type I resistance, but no significant difference in aggressiveness between the two chemotypes for Type II resistance. The study of Serajazari et al. [51] was based on inoculation of growth chamber grown plants compared to our studies using in vitro cultured spikes, the latter being under a more controlled environment and therefore being less prone to variation.

FHB severity was less in Carman-NIV infected spikes compared to those spikes infected with 3-ADON or 15-ADON producing chemotypes especially at 5, 7 and 9 DAI in agreement with a previous report that NIV producing chemotypes are less toxic to plants [52]. Analysis of variance indicated a significant effect of genotype and chemotype on FHB severity which agrees with the conclusions of Miedaner et al. [53]. The replication effect was not significant on FHB severity thus confirming the precision of the *in vitro* spike culture technique.

The five selected SCDV lines generally showed improved FHB resistance compared to Sumai-3 with the chemotypes tested. Further studies need to be conducted at the molecular level to determine expression of trichothecene biosynthetic pathway genes and other genes such as the UGT in these selected SCDV lines to further understand their patterns in response to infection by the respective chemotypes. These selected SCDV lines are currently being utilized in a breeding program to introgress the FHB resistance into elite wheat varieties.

## Supporting information

S1 Fig Chemical structures of mycotoxins selected for this study. (TIF)

S2 Fig Accumulation of Deoxynivalenol (DON), 3-Acetyldeoxynivalenol (3-ADON), 15-Acetyldeoxynivalenol (15-ADON), Nivalenol (NIV), and Deoxynivalenol-3-Glucoside (D3G) together with Fusarium Head Blight (FHB) severity at 2, 5, 7, 9, and 11 days after inoculation with *Fusarium graminearum* chemotypes (M9-07-1, M1-07-2, Carman-NIV, China-Fg809, and Carman-705-2). (TIF)
