# Supplementary figures and images for: Spike culture derived wheat (Triticum aestivum L.) variants exhibit improved resistance to multiple chemotypes of Fusarium graminearum
Source: PLoS One. 2019 Dec 19;14(12):e0226695. doi: 10.1371/journal.pone.0226695 (PMC6922434; doi:10.1371/journal.pone.0226695)

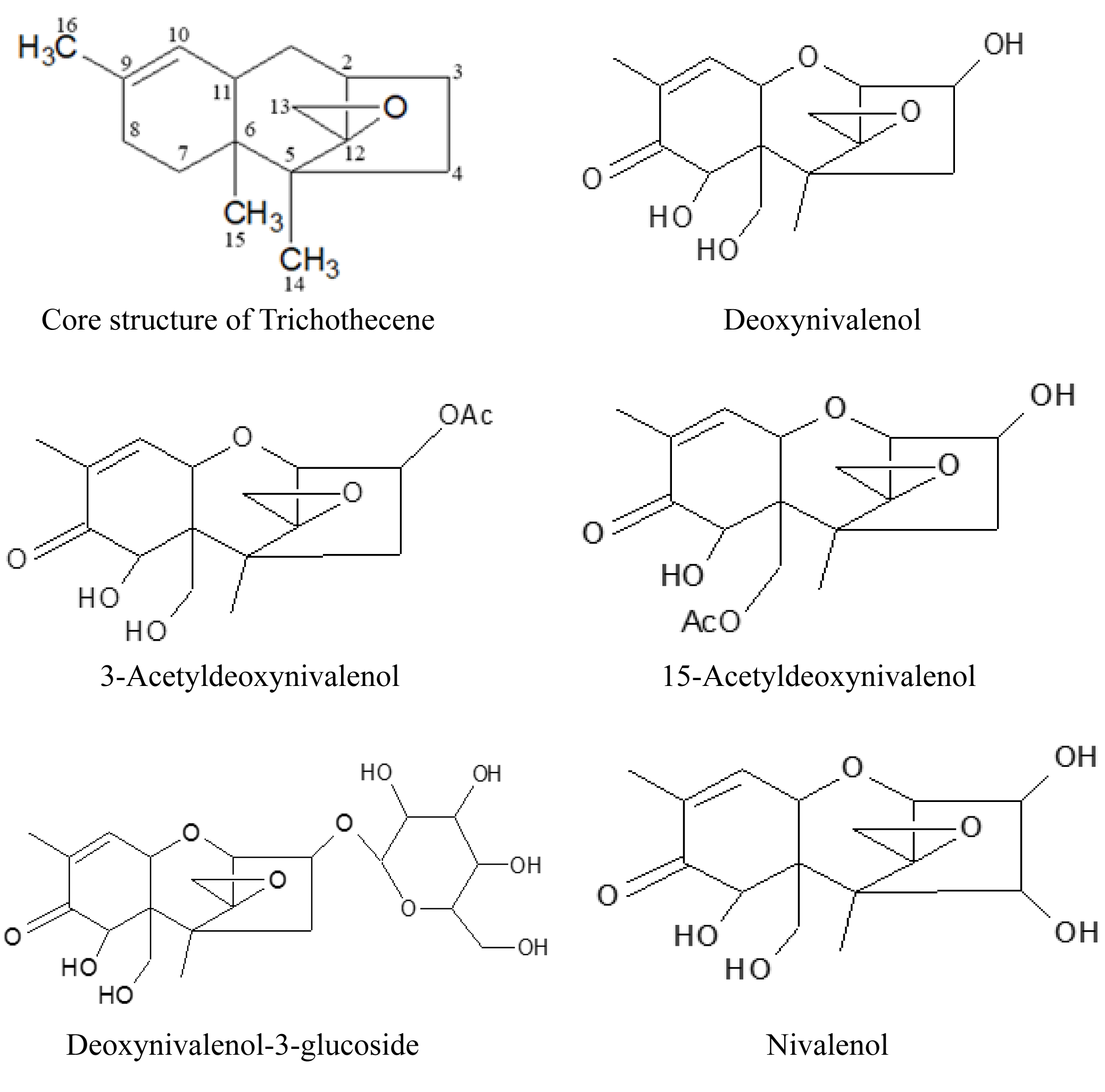

Supplement: S1 Fig — (TIF) [file pone.0226695.s001.tif]

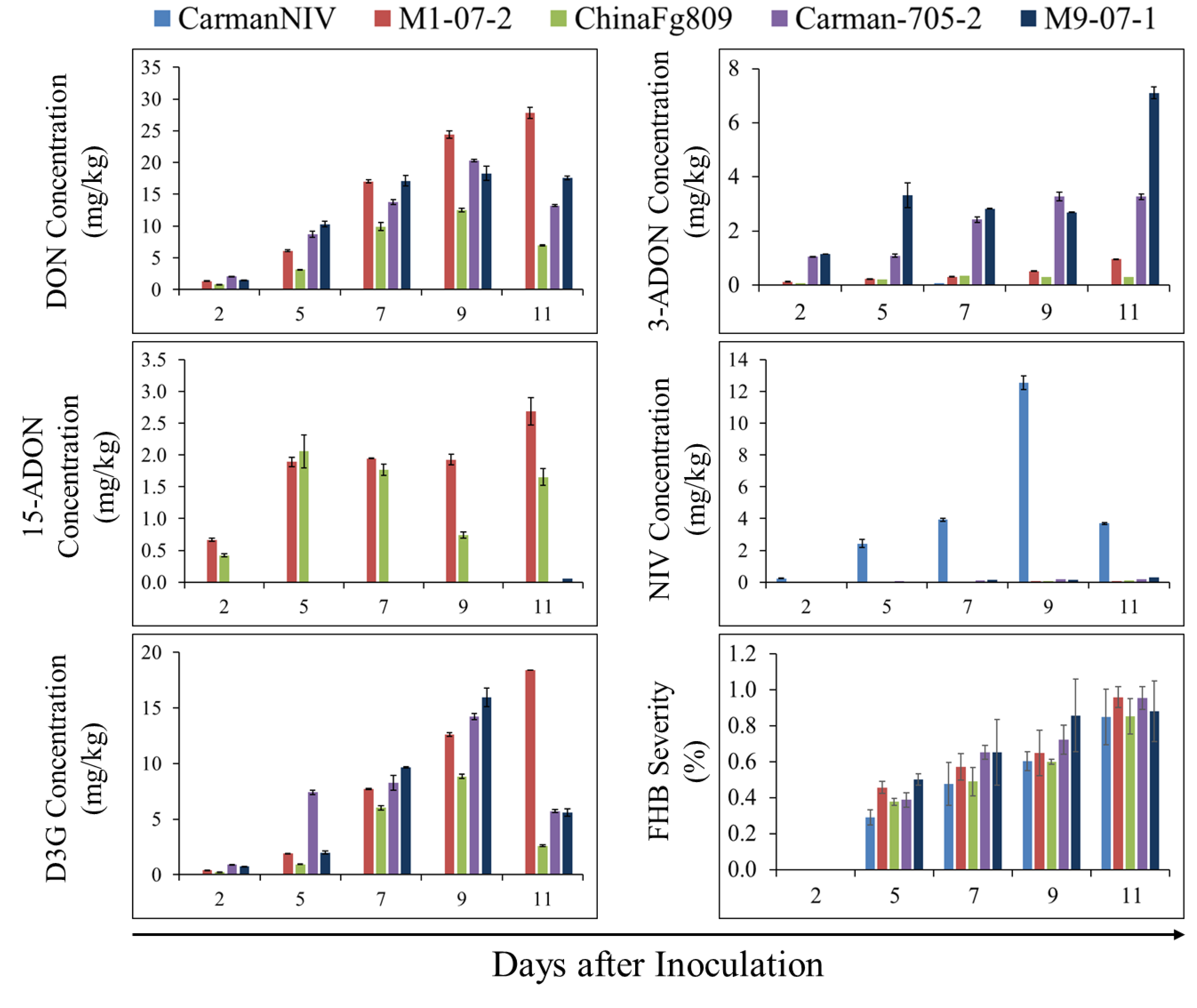

Supplement: S2 Fig — (TIF) [file pone.0226695.s002.tif]
